# Prevalence and Multi-Locus Genotyping of *Enterocytozoon bieneusi* in Dogs from Fujian Province, Southeast China

**DOI:** 10.3390/ani16060862

**Published:** 2026-03-10

**Authors:** Kai Hu, Yanlong Gu, Sheng-Jie Tang, Si-Ang Li, Yun-Peng Bai, Shang-Lin Li, Dong-Hui Zhou

**Affiliations:** 1 Key Laboratory of Fujian-Taiwan Animal Pathogen Biology, College of Animal Sciences, Fujian Agriculture and Forestry University, Fuzhou 350002, China; 2 Key Laboratory of Animal Pathogen Infection and Immunology of Fujian Province, College of Animal Sciences, Fujian Agriculture and Forestry University, Fuzhou 350002, China

**Keywords:** *Enterocytozoon bieneusi*, prevalence, dog, Fujian province

## Abstract

*Enterocytozoon bieneusi* is a microsporidian species found ubiquitously in both invertebrate and vertebrate hosts including domestic and wild animals and humans. *Enterocytozoon bieneusi* typically causes severe or chronic diarrhea, malabsorption and emaciation. This study aimed to investigate the prevalence and genotypic distribution of *E. bieneusi* in dogs in Fujian province, China. A total of 506 fecal samples from dogs were randomly collected from eight districts in Fujian province, China. The presence of *E. bieneusi* was confirmed through nested PCR targeting ITS gene. Further multilocus sequence typing (MLST) focused on the three microsatellite loci (MS1, MS3, and MS7) and minisatellite locus (MS4) loci. As a result, the infection rates of *E. bieneusi* in dogs were found to be 5.93% (30/506). A highly significant difference in the prevalence of *E. bieneusi* was observed across different urban areas (*p* < 0.01), with Longyan city exhibiting the highest infection rate (24.62%, 16/65), Zhangzhou and Xiamen the lowest (0.00%). Prevalence also varied significantly by source (*p* < 0.01), age (*p* < 0.01), gender (*p* < 0.05), symptom status (*p* < 0.01), and season (*p* < 0.01). Three known genotypes of *E. bieneusi* were identified in 30 dogs’ positive samples, including EbpC, PigEBITS5 and PtEb IX, whereas FJLYD1, FJLYD2 and FJSMD have been identified as new genotypes. EbpC, PigEBITS5, FJLYD1, FJLYD2, and FJSMD all belong to Group 1, while PtEb IX is assigned to Group 11. Genotypes belonging to Group 1, the first major phylogenetic clade, are considered to possess potential zoonotic risks. None of the positive samples amplified at all four loci, forming a single multilocus genotype (MLG). This study contributes to a deeper understanding of *E. bieneusi* in dogs, which provides critical data for the development of targeted control strategies in Fujian province.

## Simple Summary

*Enterocytozoon bieneusi* is a prevalent microsporidian pathogen infecting humans and a wide range of animal species. The characteristics of *E. bieneusi* infection are acute or chronic diarrhea, malabsorption, and emaciation. The objective of this study was to investigate the prevalence and genotypic distribution of *E. bieneusi* in dogs in Fujian province, southeast China. In this study, 506 dogs’ fecal samples were randomly collected from eight districts in Fujian province, China. We analyzed *E. bieneusi* infection rate using nested PCR targeting the internal transcribed spacer (ITS) region of the rRNA gene. Multilocus sequence typing (MLST) was performed at three microsatellite loci (MS1, MS3, and MS7) and one microsatellite locus (MS4). These results showed that 5.93% (30/506) of dog samples tested positive for *E. bieneusi*, including genotypes Ebp C, PigEBITS5, PtEb IX, FJLYD1, FJLYD2, and FJSMD. This study contributes to a deeper understanding of *E. bieneusi* in dogs, and provides critical data for the development of targeted control strategies in Fujian province.

## 1. Introduction

*Enterocytozoon bieneusi* is a common opportunistic intestinal pathogen that can infect virtually all vertebrates and invertebrates including humans and dogs, with a high degree of genetic diversity [1]. *Enterocytozoon bieneusi* occurs primarily via the fecal-oral route, through ingestion of water or food contaminated with infectious spores, or direct contact of spores with the eyes, mucous membranes, or abraded skin [2,3]. Clinical symptoms of *E. bieneusi* infection are characterized by chronic to acute diarrhea, malabsorption, weight loss, wasting, diffuse abdominal pain and acalculous cholecystitis, etc., and even life-threatening diarrhea in immunocompromised individuals [4,5].

To date, more than 685 genotypes of *E. bieneusi* have been identified in humans and animals (including cattle, pigs, dogs, cats and birds, etc.) based on sequence analysis of the ribosomal internal transcribed spacer (ITS) [6]. These genotypes are categorized into 11–13 phylogenetic groups that display varying degrees of host specificity and zoonotic potential [6]. Group 1 is the largest group, further divided into nine subgroups, and contains most zoonotic genotypes including D, EbpC and Type IV, etc., which are commonly detected in humans. Group 2 is the second largest cluster, divided into three subgroups which are the most frequent contributors to *E. bieneusi* infection in cattle, deer, sheep, and goats, including BEB4, BEB6, I, and J. Groups 3–11 contain fewer host-adapted variants or unknown zoonotic significance and are not further subdivided [5,6].

The ITS-based genotyping is often unable to present subtle changes needed for subgrouping and accordingly might not adequately represent the evolutionary history of this pathogen. Multilocus sequence typing (MLST)-based phylogenetic analysis is more discriminant and identifies within Group 1 seven subpopulations (SP) presenting host specificities, whereas they were not distinguished within ITS-based subgroups. The MLST-based population genetic analysis would be more precise in assessing subpopulation formation and host-specific differences [4,5,6,7,8].

Dogs are one of the most important companion animals to humans and are considered important infection sources of zoonotic pathogens including *E. bieneusi*. The few studies about *E. bieneusi* in dogs conducted so far showed that the infection rates were 0.8–31.3%, and several zoonotic ITS genotypes—including the most common zoonotic genotypes, Type IV and D—have been identified [5,9,10,11,12]. However, the prevalence and genotype of *E. bieneusi* in dogs in Fujian province have not been reported. This study was conducted on molecular detection and genotype identification of *E. bieneusi* in dogs from Fujian province, southeast China. These findings offer foundational data for the prevention and control of *E. bieneusi* in humans and dogs in this area, and provide a reference for public health preparedness and control under the One Health framework.

## 2. Materials and Methods

### 2.1. Sample Collection and Processing

Between September 2020 and March 2022, 506 canine-derived samples were collected from a total of 74 sampling sites (36 pet hospitals, 33 pet shops, 4 stray animal shelters and 1 farm) in 7 administrative districts in Fujian province, including 477 normal fecal samples and 29 diarrhea samples. Sample age classification included 268 samples from dogs aged ≤1 year old, and 238 samples from those > 1 year old. The age information on the dogs is provided by the dogs’ owners. In terms of gender, there are 248 male samples and 258 female samples. The fecal samples were collected according to the following seasons and their corresponding months: spring (February–April), summer (May–August), autumn (September–October), and winter (November–January).

Samples were collected using medical sterile collection tubes, sealed to prevent cross-contamination and labeled with information regarding region, gender and age. These samples were subsequently refrigerated and transported to the laboratory, where they were stored in 2.5% potassium dichromate solution and kept in a refrigerator at 4 °C until further analysis.

### 2.2. Extraction of Fecal Genomic DNA

All samples were diluted with distilled water, mixed thoroughly, and centrifuged. The supernatant containing potassium dichromate was discarded, and the procedure was repeated until the upper layer was clear. Approximately 200 mg of feces was transferred to a 2 mL centrifuge tube. Distilled water was added to the feces and mixed, which were then placed in boiling water at 100 °C for 5 min. They were then transferred to a refrigerator at −80 °C for 5 min and subjected to five freeze–thaw cycles. Finally, DNA was extracted according to the E.Z.N.A^®^ Stool DNA Kit instructions (Omega Biotek Inc., Norcross, GA, USA).

### 2.3. Genotype Identification

The primers were designed targeting the internal transcribed spacer (ITS) region of the small subunit ribosomal RNA (SSU rRNA) gene from *E. bieneusi*, and genotype identification was performed through nested PCR amplification. The primer sequences and amplification information for the nested PCR reaction are provided in Table 1.

MLST was performed using the *E. bieneusi* MLST scheme established by Feng et al. [13], targeting three microsatellite loci (MS1, MS3, MS7) and one minisatellite locus (MS4) in ITS-positive samples.

### 2.4. Sequencing and Phylogenetic Analysis

The amplification products were analyzed by 1.5% (*w*/*v*) agarose gel electrophoresis. The positive second-round PCR products were sent to Beijing Tsingke Biotech Co., Ltd., Beijing, China for two-directional sequencing. Bioinformatics software DNASTAR 7.1 (https://www.dnastar.com/, accessed on 20 November 2025) was applied to splice and correct the sequencing results, and the sequences were compared with Jalview (http://www.jalview.org/, accessed on 21 November 2025). The aligned and spliced nucleotide sequences were compared online using BLAST+ 2.17.0 in NCBI (http://www.ncbi.nlm.nih.gov, accessed on 22 November 2025) to determine the genotypes and polymorphisms of *E. bieneusi*. Phylogenetic analysis was conducted in MEGA 11; sequence alignment was performed using the Clustal W algorithm, and the evolutionary tree was constructed using the Neighbor-Joining method based on the Kimura 2-parameter (K2P) genetic distance model with node support evaluated based on 1000 bootstrap replicates.

### 2.5. Statistical Data Analysis

Statistical analysis of *E. bieneusi* infection rates in dogs by region, source, age, gender, and presence of diarrhea was performed in SPSS 26.0 (SPSS, Inc., Chicago, IL, USA) software. The chi-square test was used for intergroup difference analysis when the expected count in the contingency table was ≥5; Fisher’s exact test was applied instead when the expected count was <5. Differences between groups were considered statistically significant when *p* < 0.05.

## 3. Results

### 3.1. Prevalence of E. bieneusi in Dogs

A total of 30 positive samples were identified among 506 canine-derived feces from seven regions in Fujian province, yielding an overall detection rate of 5.93% (Table 2). The highest prevalence was observed in Longyan City 24.62% (16/65), followed by Fuzhou City at 5.83% (8/137) and Quanzhou City at 2.83% (3/106).

There was also a highly significant difference in *E. bieneusi* prevalence among dogs of different origins (χ^2^ = 33.134, *p* < 0.01), with infection rates ranging from 2.33% to 18.68%; dogs from stray animal shelters had the highest infection rate (18.68%) while those from veterinary hospitals had the lowest (2.33%). For dogs of different ages, the infection rate of *E. bieneusi* ranged from 2.10% to 9.32%, with the highest positive rate in dogs aged ≤ 1 year (9.32%) and the lowest in those aged > 1 year (2.10%), showing a highly significant statistical difference (χ^2^ = 11.806, *p* < 0.01), indicating age may be a key factor influencing the infection rate. A significant difference in *E. bieneusi* prevalence was found between male and female dogs (χ^2^ = 3.978, *p* < 0.05), with the positive rate at 8.06% in males and 3.88% in females. Among the 506 samples, the positive rate of *E. bieneusi* in diarrheic samples reached 31.03%, in sharp contrast to only 4.40% in non-diarrheic samples, with a highly significant statistical difference (χ^2^ = 34.765, *p* < 0.01). In terms of seasonal variation, the positive rate was 3.93% in spring, 12.42% in summer, 3.36% in autumn and 1.87% in winter, showing a highly significant difference across seasons (χ^2^ = 17.024, *p* < 0.01).

### 3.2. Analysis of E. bieneusi Genotype and MLST

Thirty positive samples were subjected to sequencing, and the resulting sequences were spliced and entered into a BLAST+ 2.17.0 search. The sequences were then analyzed for germline development with reference sequences from GenBank. The present study identified six genotypes, of which EbpC, PigEBITS5, and PtEb IX were already known, whereas FJLYD1, FJLYD2, and FJSMD were identified as new genotypes. PtEb IX was the most prevalent genotype of *E. bieneusi* infection in dogs, which shows a widespread distribution across all regions. The PtEb IX genotype was detected in dogs from veterinary hospitals, pet stores, farms and stray animal shelters. Its wide distribution in different environments indicates its potential for transmission and persistence. Notably, five genotypes (PtEb IX, EbpC, FJSMD, FJLYD1, FJLYD2) were identified in dogs aged ≤ 1 year, while only the PtEb IX genotype was detected in dogs aged > 1 year. This difference in genotype distribution suggests that there may be age-related susceptibility patterns or transmission dynamics in dogs. Four genotypes were detected in both genders, with PtEb IX and EbpC being the common genotypes. FJLYD1 and FJLYD2 were only found in males, while PIGEBITS5 and FJSMD12 were only found in females. The PtEb IX genotype was consistently detected in positive samples from all four seasons (Table 3).

A total of 506 dog fecal samples were collected, of which 30 tested positive for the *E. bieneusi* ITS gene. Two MS1 locus positive samples, three MS3 positive samples, and two MS7 positive samples were identified, but no positive samples were detected for the MS4 locus (Table 4).

### 3.3. Phylogenetic Analysis of E. bieneusi in Dogs

**Figure 1:**
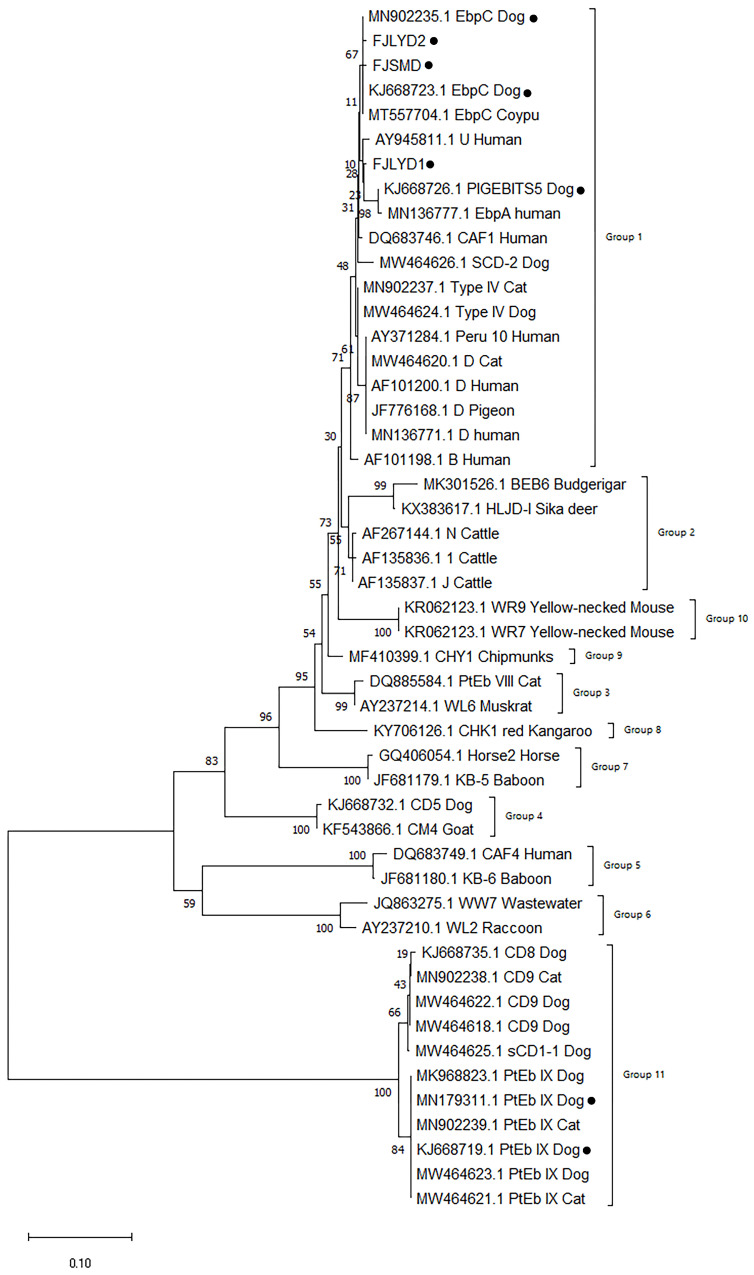
Phylogenetic tree of genotypes of *Enterocytozoon bieneusi* genotypes based on ITS sequence. The Kimura 2 parameter method was used with bootstrap evaluation of 1000 replicates. Solid black circles represent the genotypes of positive samples identified in our laboratory.

An evolutionary tree of the ITS genes was constructed using the ClustalW for sequence alignment and the NJ method. These results demonstrated that the genotypes of EbpC, PigEBITS5, FJLYD1, FJLYD2, and FJSMD were classified within Group 1, whereas PtEb IX was assigned to Group 11. The first seven and the latter were identified as belonging to two distinct groups. The relationships between these sequences were found to be distantly related. FJLYD1 and FJLYD2 were found to be on the same level as the reference sequences (EbpC) and exhibited close affinity. FJSMD was found to be on the same branch as PigEBITS5 and had the closest affinity (Figure 1).

Three novel genotypes were identified based on ITS locus sequencing of the 30 *E. bieneusi*-positive samples; their corresponding DNA sequences have been deposited in the GenBank database under accession numbers PZ101889–PZ101891.

## 4. Discussion

*Enterocytozoon bieneusi* is one of the most epidemiologically significant microsporidian pathogens, with confirmed infections reported in a diverse array of vertebrate and invertebrate hosts. Cumulative evidence indicates its presence in at least 210 terrestrial mammalian and avian species, including dogs. *Enterocytozoon bieneusi* is widely recognized as an important opportunistic pathogen causing diarrhea in both immunocompromised and immunocompetent individuals [5,14]. Although its infection usually does not lead to acute fatal outcomes, recent epidemiological surveillance data show that the detection rate and disease burden of this pathogen have been continuously increasing in recent years, highlighting its growing public health significance [15].

As one of the most intimate companion animals of humans, dogs have been recognized as important animal hosts of various zoonotic pathogens due to their long-term and frequent direct contact with humans [16,17]. Therefore, within the One Health framework, establishing a collaborative prevention and control system for veterinary and public health, systematically monitoring canine health data, and prioritizing targeted investigations of zoonotic pathogens with undefined regional prevalence have become key tasks to safeguard the holistic health of humans, animals, and the environment. In this study, the overall infection rate of *E*. *bieneusi* from dogs was 5.93%, and the infection rates ranged from 0% to 24.6% in Fuzhou, Ningde, Sanming, Longyan, Zhangzhou, Quanzhou, and Xiamen, which may be related to the environments where the dogs were living and the public hygiene conditions. Compared with the prevalence of *E. bieneusi* in other countries, the prevalence in this study was lower than that in observed in South Korea (14.2%), Malaysia (40.7%), and Thailand (24.3%) and the regions of Sichuan (18.8%), Henan (13.9%), Jilin (7.8%), and Anhui (9.3%) in China [10,18,19,20,21,22,23]. However, they were higher than reported infection rates in Japan (4.4%), Iran (5.3%), Spain (5.0%), Zhejiang (7.0%), Heilongjiang (6.7%) [22], and Xinjiang (6.3%) [24]. This may be due to climate. Studies have shown that the prevalence of *E. bieneusi* is relatively high in European countries. The results of the climate subgroup indicate that the disease incidence is highest in temperate marine climates. Research shows that extreme temperatures and precipitation are not conducive to the growth of *E. bieneusi* [25,26]. The temperate maritime climate has the characteristics of warm winters and cool summers, which are characterized by small annual temperature variations and even precipitation distribution. This may be beneficial for the growth and spread of *E. bieneusi*. Therefore, differences in infection rates may be related to geography, ecological environment, host immunity, personal hygiene, cultural norms, and testing methods.

Dogs, as companion animals, cohabit closely with humans and other animals, rendering them susceptible to a wide range of zoonotic pathogens. Among dogs from different sources, those from shelters had the highest prevalence 18.7% (17/91) and those from pet hospitals with owners had the lowest prevalence 2.3% (6/257), with highly significant differences in the prevalence of infection among dogs from different sources (*p* < 0.01). The results of this study are consistent with the results of testing for *E. bieneusi* infection in Yunnan province, Shanghai, and Japan [12,20,27]. The high positive rate of dogs in shelters may be related to cross infection, as there are too many dogs living in poor environments because they are not restrained and live in groups in captivity. The detection rate of *E. bieneusi* in dogs presented at pet hospitals is relatively low, likely attributable to their favorable living conditions and routine anthelmintic treatment. In contrast, stray dogs exhibit a higher infection risk, highlighting their potential role as vectors of zoonotic diseases and the associated public health concerns involving human, animal, and environmental health.

Correlation analysis shows that the incidence of *E. bieneusi* infection in puppies is significantly higher than that in adult dogs (9.3% vs. 2.1%). Similar findings have been reported in epidemiological surveys of *E. bieneusi* infection across multiple regions, including Xinjiang (China), Japan, Australia, and Romania [18,28]. These findings further substantiate the association between young age and increased susceptibility to *E. bieneusi* infection. Additionally, a notable difference in infection rates was observed between male and female dogs (*p* < 0.05). But the test results are opposite to those in Guangzhou, Xinjiang, Colombia, and Romania [18,21,24,29]. In addition, this study found that *E. bieneusi* infection was significantly positively correlated with diarrhea symptoms in dogs.

The 30 positive samples were identified on the following dates: 28 April, 14 July, 4 September, and 6 October. The results demonstrated that the disease has the potential to manifest throughout the year, with a markedly pronounced disparity in the positivity rate across different seasons (*p* < 0.01). The higher positivity rate in summer may be related to the fact that the parasite is relatively more active and easily transmitted in the summer due to the high rainfall and hot weather, which facilitates contact between dogs and *E. bieneusi* spores, leading to the occurrence of the disease.

Through sequence alignment analysis, six *E. bieneusi* genotypes were identified in dogs in this study: EbpC, PigEBITS5 and PtEbIX are known genotypes; FJLYD1, FJLYD2 and FJSMD are newly reported genotypes for the first time. Among them, five genotypes (including all three new genotypes) belong to the Group 1 evolutionary branch, with clear zoonotic potential. Given that all known genotypes within this evolutionary branch have been confirmed or highly suspected to be capable of infecting humans, the newly discovered FJLYD1, FJLYD2 and FJSMD in this evolutionary branch provides indirect phylogenetic evidence for their potential zoonotic risk, and these novel genotypes are therefore considered as putative zoonotic pathogens that require further validation of their transmission capacity between humans and animals. Among the 30 positive samples obtained from dogs, genotype PtEb IX was predominant, which is a dog-specific genotype. It accounted for 70% (21/30) of the total number of all positive samples, which was consistent with the reports from Anhui and Zhejiang regions in China [23], Yunnan [27], and Xinjiang [24]. The EbpC genotype (13.3%, 4/30) is widely present in a wide range of hosts in humans, pigs, sheep, horses, rabbits, and non-humans primates [9]. The EbpC genotype was reported in vegetables, fruits, and sewage in Shanghai, Nanjing, Wuhan, Qingdao, and Zhengzhou in China [30,31,32]. Furthermore, EbpC is the main genotype of microsporidiosis in Harbin children and Henan patients with AIDS [33]. Thus, dogs, as an important companion animal in close contact with humans, are one of the important sources of human microsporidiosis infection and water contamination in China, which are hazardous to humans health and public health safety.

Although this study systematically analyzed the infection status and public health risks of *E*. *bieneusi* in dogs in Fujian province, it still has several limitations. First, this study only focused on canines and did not collect human samples for homology comparison or analysis of *E. bieneusi* infection. Second, the MLST successful amplification rate of some isolates was limited, and complete sequence typing results were not obtained, which restricted further analysis of the population structure, genetic evolutionary relationship, and potential transmission routes of the isolates to a certain extent. The main reason may be attributed to the presence of PCR inhibitors in the fecal samples, which seriously affect the quality of the DNA template and the activity of the polymerase [34,35,36]. In addition, nucleases from the microorganisms in the samples may degrade the template DNA and primers, leading to amplification failure [34]. Finally, the sample size of some subgroups in the study was small; for example, the number of diarrheic dogs was only 29, accounting for a low proportion compared with the total sample size (*n* = 506). This may lead to inaccurate statistical results regarding the difference in infection rates between subgroups.

This study successfully amplified 30 ITS positive samples, which were amplified at three loci of MS1, MS3 and MS7 microsatellites, respectively, generating 3, 4 and 2 positive samples. None of positive sample amplified at all four loci, forming a single multilocus genotype (MLG). Through MLST analysis of *E. bieneusi* genotypes, the possibility of co-infection between humans and animals can be evaluated to trace the source of infection and clearly understand the transmission mechanism.

## 5. Conclusions

This study analyzed 506 dog feces from Fujian, with a dog detection rate of 5.93%. Three known genotypes of *E. bieneusi* were identified in 30 dogs’ positive samples, including EbpC, PigEBITS5 and PtEb IX, whereas FJLYD1, FJLYD2 and FJSMD have been identified as new genotypes. EbpC, PigEBITS5, FJLYD1, FJLYD2, and FJSMD all belong to Group 1, while PtEb IX is assigned to Group 11. Genotypes belonging to Group 1, the first major phylogenetic clade, are considered to possess potential zoonotic risks. This phylogenetic placement suggests that the novel genotypes have putative zoonotic risk, though direct evidence for their zoonotic transmission remains to be investigated in future studies. There is an urgent need for effective strategies to reduce cross infection between animals and humans, thereby strengthening overall disease prevention and public health protection.

## Figures and Tables

**Table 1:** The primer used in this study.

Primers	Sequences (5′-3′)
F1	GGTCATAGGGATGAAGAG
R1	TTCGAGTTCTTTCGCGCTC
F2	GCTCTGAATATCTATGGCT
R2	ATCGCCGACGGATCCAAGTG

**Table 2:** Prevalence of *E. bieneusi* infection in Fujian dogs.

Factors	Category	No. Sample	No. Positive Rate (95% CI)
Area	Fuzhou	137	8 (5.83%, 2.82–11.87%)
	Longyan	65	16 (24.62%, 15.67–36.40%)
	Ningde	46	1 (2.17%, 0.01–12.38%)
	Quanzhou	106	3 (2.83%, 0.61–8.35%)
	Sanming	76	2 (2.63%, 0.17–9.65%)
	Zhangzhou	32	0 (0%, 0.00–12.73%)
	Xiamen	44	0 (0%, 0.00–9.58%)
Source	Pet Hospital	257	6 (2.33%, 0.95–5.12%)
	Pet Shop	116	5 (4.31%, 1.60–9.95%)
	Farms	42	2 (4.76%, 0.46–16.65%)
	Shelter	91	17 (18.68%, 11.91–27.99%)
Age	≤1 year old	268	25 (9.32%, 6.35–13.45%)
	>1 year old	238	5 (2.10%, 0.76–4.96%)
Gender	Male	248	20 (8.06%, 5.22–12.19%)
	Female	258	10 (3.88%, 2.03–7.08%)
Diarrhea	yes	29	9 (31.03%, 17.14–49.37%)
	No	477	21 (4.4%, 2.86–6.67%)
Season	Spring	127	5 (3.93%, 1.45–9.12%)
	Summer	153	19 (12.42%,8.02–18.66%)
	Autumn	119	4 (3.36%, 1.03–8.61%)
	Winter	107	2 (1.87%, 0.10–6.98%)
	Total	506	30 (5.93%, 4.16–8.16%)

**Table 3:** Prevalence of *E. bieneusi* genotypes in dogs in Fujian province.

Factors	Category	Genotype (No.)
Area	Fuzhou	PtEb IX(8)
	Longyan	PtEb IX(9), Ebp C(4), FJLYD 1(2), FJLYD 2(1)
	Ningde	PtEb IX(1)
	Quanzhou	PtEb IX(3)
	Sanming	FJSMD 12 (1), PIGEBITS 5 (1)
	Zhangzhou	
	Xiamen	
Source	Pet Hospital	PtEb IX(5), EbpC(1)
	Pet Shop	PtEb IX(5)
	Farms	PtEb IX(1), FJSMD(1)
	Shelter	PtEb IX(11), FJLYD1(2), FJLYD2(1), EbpC(3)
Age	≤1 year old	PtEb IX(16), EbpC(4), FJLYD1(2), FJLYD2(1), FJSMD (1), PIGEBITS 5 (1)
	>1 year old	PtEb IX(5)
Gender	Male	PtEb IX(14), EbpC(3), FJLYD1(2), FJLYD2(1)
	Female	PtEb IX(7), EbpC(1), PIGEBITS5(1), FJSMD12(1)
Diarrhea	yes	PtEb IX(8), EbpC(1)
	No	PtEb IX(13), EbpC(3), FJLYD1(2), FJLYD2(1), PIGEBITS5(1), FJSMD(1)
Season	Spring	PtEb IX(5)
	Summer	PtEb IX(13), FJLYD1(2), FJLYD2(1), FJSMD(1), EbpC(1)
	Autumn	EbpC(3), PtEb IX(1), PIGEBITS5(1)
	Winter	PtEb IX(2)

**Table 4:** MLST genotypes of *E. bieneusi*.

Isolated Strain	Genotype	MS 1	MS 3	MS 4	MS 7	MLGs
FZ-32	Pt EbIX	Type 1	Type 1	-	-	-
FZ-54	Pt EbIX	-	Type 1	-	Type 1	-
QZ-33	Pt EbIX	Type 1	Type 1	-	-	-
SM-29	PIGEBITS 5	-	-	-	Type 1	-

Note: Type I represents allele type identified at each microsatellite locus based on sequence polymorphisms compared to reference sequence.

## Data Availability

The original contributions presented in this study are included in the article.
